# World Pneumonia Day — November 12, 2013

**Published:** 2013-11-08

Every 20 seconds, somewhere in the world, a child dies from pneumonia (1). Many of these deaths are preventable through appropriate treatment and vaccination. With support from the GAVI Alliance, notable progress has been made in preventing pneumonia deaths and hospitalizations resulting from *Streptococcus pneumoniae* (pneumococcus) and *Haemophilus influenzae* type b (Hib) infections (2,3).

In spring 2013, the World Health Organization and the United Nations Children’s Fund (UNICEF) released the Global Action Plan for Pneumonia and Diarrhoea, which promotes pneumococcal conjugate vaccine use as an important strategy for achieving United Nations Millennium Development Goal 4 to reduce child mortality (4). Hib conjugate vaccine also is becoming a part of global routine infant immunization, and recent data show its effectiveness at preventing pneumonia in developing countries (2,4).

In spite of this progress, many gaps remain. Respiratory viruses, such as respiratory syncytial virus, influenza, and measles, also are major causes of pneumonia globally. Expanded use of influenza and measles vaccines, antiviral medications, and supportive health care can reduce the burden of pneumonia caused by these viruses. Additional research on diagnostics, prevention, and treatment of viral-associated pneumonia also is needed.

World Pneumonia Day is being observed November 12, 2013, to raise awareness about pneumonia’s toll and to promote interventions to protect against, treat, and prevent the disease globally. Activities are being promoted by a coalition of more than 140 community-based organizations, academic institutions, government agencies, and foundations. More information is available at http://worldpneumoniaday.org.
